# P-684. Predictors and Outcome of Hospital-acquired Pneumonia and Ventilator-associated Pneumonia Secondary to Multidrug-resistant Pathogens among Intensive Care Unit Patients

**DOI:** 10.1093/ofid/ofaf695.897

**Published:** 2026-01-11

**Authors:** Aishath Razana Rasheed, Arun Shahi, nora Ranjitkar, janak koirala

**Affiliations:** Patan Academy of Health Sciences, Lalitpur, Bagmati, Nepal; Patan Academy of Health Sciences, Lalitpur, Bagmati, Nepal; patan Academy of Health Sciences, Lilitpur, Bagmati, Nepal; Patan Academy of Health sciences, lalitpur, Bagmati, Nepal

## Abstract

**Background:**

Hospital-acquired pneumonia (HAP) and ventilator-associated pneumonia (VAP) contribute substantially to morbidity and mortality in the intensive care units (ICU). This has been exacerbated by rising antimicrobial resistance (AMR) in low-resource regions. This study aimed to identify the predictors for HAP and VAP caused by multi-drug resistant (MDR) gram-negative rods (GNR) among ICU patients and to assess outcomes.

**Methods:**

A prospective cohort study was conducted from September 2024 to April 2025 at Patan Academy of Health Sciences, Nepal. All patients over 14 years admitted in ICU and meeting diagnostic criteria for HAP or VAP were enrolled and followed until discharge. Predictive factors for MDR GNR infection were analyzed including prior intravenous (IV) antibiotic use within 90 days, patient rooming proximity to known cases of MDR GNR, disease severity at admission, and septic shock at presentation. Outcomes including length of hospital stay and mortality were compared between patients with MDR and non-MDR GNR infections.

**Results:**

Among 80 ICU patients, 75% had HAP and 25% had VAP. Prior IV antibiotic use within 90 days was associated with a 4.3-fold increased risk (p=0.002), while rooming adjacent to a known case of MDR GNR conferred a 3.5-fold higher risk (p=0.007). Patients presenting with septic shock at baseline had a 7.4-fold increased likelihood of MDR GNR infection (p=0.001). Median baseline SOFA score of patients with MDR GNR infection was 7 (IQR 4-10) compared to 4 (IQR 2-4.75) for Non-MDR infection (p< 0.001). The mortality rate was substantially higher in the MDR group (42.5%) compared to non-MDR group (7.5%) (OR 9.1, p< 0.001). Duration of hospital stay was significantly longer for patients with MDR infection (24 vs. 10 days, p< 0.001).

**Conclusion:**

Among ICU patients with HAP/VAP, presence of septic shock and higher SOFA score at presentation, recent antibiotic exposure, and close proximity to patients with MDR GNR were factors associated with MDR pathogens. HAP/VAP secondary to MDR GNR was associated with increased mortality and duration of hospitalization, underscoring the need for judicious use of antibiotics and better infection control practices including maintaining distance for patient rooming.

**Disclosures:**

All Authors: No reported disclosures

